# Amanita muscaria in the evolving novel psychoactive substances landscape - toxicological risks and clinical implications: a narrative review

**DOI:** 10.3389/fphar.2026.1838212

**Published:** 2026-05-26

**Authors:** Michal Ordak

**Affiliations:** Centre of Regenerative Medicine, Medical University of Bialystok, Bialystok, Poland

**Keywords:** *Amanita muscaria*, fly agaric, ibotenic acid, muscimol, new psychoactive substances

## Abstract

The rapid evolution of the novel psychoactive substances market has blurred the boundaries between synthetic “designer drugs” and other emerging psychoactive products distributed outside traditional regulatory frameworks. Recently, increasing recreational and self-therapeutic interest in *Amanita muscaria* has led to its growing presence within the broader landscape of substances used in patterns similar to novel psychoactive substances. Widely available through online platforms and social media, it is promoted for stress reduction, mood improvement, sleep enhancement, and analgesic purposes, despite limited clinical evidence and established toxicity risks. This narrative review synthesizes available clinical and toxicological evidence on *A. muscaria* and its reported adverse health outcomes. Emerging reports indicate increasing intentional consumption, heterogeneous dosing practices, and presentations ranging from mild neurological symptoms to severe intoxication requiring hospitalization. Online narratives and informal harm reduction advice contribute to normalization of use and potential underestimation of risks. Within the context of novel psychoactive substances research, *A. muscaria* represents a regulatory and public health challenge: a non-synthetic psychoactive substance integrated into digital drug markets and self-experimentation cultures. The absence of standardized management guidelines and systematic surveillance complicates clinical response. Greater awareness of its toxicological profile, patterns of misuse, and emergency management strategies is needed to inform clinicians, toxicologists, and public health authorities.

## 1 Introduction

Since the mid-2000s, numerous substances marketed as legal alternatives to controlled illicit drugs have emerged and are collectively referred to as new psychoactive substances (NPS). Their rapid appearance and diversity have complicated drug monitoring, regulation, and public health responses while reflecting changes in drug markets and user motivations. Evidence shows that the availability, use, and harms associated with NPS highlight the need for improved systems to detect, identify, and respond to emerging substances (Peacock et al., 2019). Further research is needed to more accurately characterize the pharmacological effects, toxicological risks, and public health implications associated with emerging NPS. Strengthening analytical detection methods and expanding knowledge of their mechanisms of action are essential for improving public health responses and guiding future research on these rapidly evolving substances (Marandiuc et al., 2025).

In recent years, the implementation of legal regulations controlling NPS has limited the availability of many of these substances on the drug market. As a result, some users have turned to more easily accessible alternatives, including ephedrone, also known as methcathinone, which can be synthesized from medications containing pseudoephedrine (Ordak, et al., 2022; Wang et al., 2025). To address this issue, measures limiting the amount of over-the-counter medications containing pseudoephedrine that can be purchased in a single transaction have been introduced in certain regions. According to studies conducted in Western Australia, the implementation of a real-time pharmacy monitoring system for pseudoephedrine sales was associated with a reduction in product requests and proved effective in identifying suspicious transactions and limiting the potential diversion of these medications (Hattingh et al., 2016).

More broadly, when legal restrictions restrict access to certain psychoactive substances, users may seek alternative compounds capable of producing similar psychoactive effects. In some cases, this shift may involve substances that are easier to obtain and remain outside strict regulatory control, such as ephedrone (Ordak, et al., 2022). In this context, individuals seeking psychoactive experiences may increasingly turn to naturally occurring alternatives that are easier to obtain and are not always subject to the same legal restrictions as synthetic psychoactive drugs, such as *Amanita muscaria*, a psychoactive mushroom whose effects are primarily associated with muscimol and ibotenic acid. The growing interest in this species reflects a broader tendency to seek accessible psychoactive substances in response to changes in drug policies and market availability. A 2023 report by the European Food Safety Authority highlighted growing concerns about the increasing popularity of alcohol-replacement products made from edible plant materials that affect the gamma-aminobutyric acid (GABA) system, while also identifying rising interest in *A. muscaria* as a potential public health concern (Gkrintzali et al., 2024).

Although *A. muscaria* is not a synthetic NPS in the traditional sense, its contemporary patterns of promotion, online commercialization, self-experimentation, and extra-medical use resemble those observed for many novel psychoactive substances. In this context, *A. muscaria* shares several characteristics with substances that circulate within contemporary NPS markets. These include widespread promotion through social media and online platforms, informal and highly variable dosing practices shared among users, user-generated harm reduction advice not supported by clinical evidence, and therapeutic claims lacking scientific validation. This narrative review summarizes the current clinical and toxicological evidence regarding *A. muscaria*, with particular attention to patterns of use, reported adverse effects, and emerging public health concerns. The manuscript also discusses its increasing presence in online markets and self-experimentation communities, highlighting the challenges it poses for toxicological monitoring, clinical management, and regulatory frameworks.

## 2 Methods

This narrative review was conducted to summarize current clinical, toxicological, and public health evidence regarding *A. muscaria*. A comprehensive literature search was performed using the PubMed, Web of Science, and Scopus databases. The literature search included studies published through March 2026. The search strategy used combinations of the following keywords and Boolean operators: (“Amanita muscaria” OR “fly agaric”) AND (“ibotenic acid” OR “muscimol” OR “muscarine” OR “Amanita muscaria poisoning” OR “mushroom intoxication” OR “psychoactive mushrooms” OR “toxicology”). The review incorporated a range of publication types reflecting the heterogeneous nature of the available evidence, including original research articles, clinical case reports, observational studies, toxicological analyses, review articles, and public health reports. No restrictions were imposed on study design, provided that they directly addressed the pharmacology, toxicology, patterns of use, or health effects associated with *A. muscaria*. Conference abstracts, duplicate publications, and studies not directly related to *A. muscaria* were excluded. Only articles published in English were included. Studies were screened according to title, abstract, and full-text relevance to the predefined thematic areas of the review. Extracted data included study design, toxic compounds, clinical manifestations, exposure patterns, treatment approaches, and public health implications. Data were grouped according to predefined thematic domains, including pharmacological mechanisms, clinical manifestations, patterns of use, adverse effects, treatment approaches, and public health implications. The retrieved literature was synthesized narratively, with emphasis on common clinical manifestations, mechanisms of toxicity, emerging patterns of use, and potential public health risks associated with the growing popularity of *A. muscaria*. Because of the heterogeneous nature of the available evidence, a quantitative synthesis was not performed. Grey literature and user-generated online content were not included as formal sources of evidence, however selected observations from online discussion platforms were considered narratively to contextualize emerging patterns of recreational use, self-medication practices, and informal harm-reduction behaviors. The available evidence consisted predominantly of case reports, observational studies, and toxicological analyses, while high-quality prospective clinical studies remained limited.

Table 1 presents the thematic domains of the reviewed literature, the types of evidence included, and the main topics addressed in the narrative synthesis.

**TABLE 1:** Thematic domains, types of evidence, and main observations included in the narrative review of *Amanita muscaria*.

Thematic domain	Type of evidence	Main observations
Pharmacology	Experimental studies	Muscimol acts as a GABA receptor agonist
Clinical toxicity	Clinical case reports	Hallucinations, agitation, coma, and central nervous system depression
Recreational use	Social media analyses	Self-medication practices and recreational use motivations
Preparation methods	Online forums and reddit discussions	Drying, decarboxylation, and extract preparation practices
Public health concerns	Review articles and public health reports	Increasing online popularity and emerging public health concerns

## 3 Chemical composition and pharmacological mechanisms of Amanita muscaria

Various pharmacologically active constituents present in *Amanita muscaria* may elicit a broad spectrum of neuropsychiatric and physiological manifestations, with outcomes varying according to the quantity ingested, mode of administration, and personal biological variability. *Amanita muscaria* is regarded as a toxic species because it contains several neuroactive compounds, including muscarine, ibotenic acid, and muscimol, which are responsible for its physiological and neurological effects. The first of these compounds, muscarine, is present in *A. muscaria* only in extremely low quantities, accounting for roughly 0.0003% of its weight, a level considered insufficient to produce toxic effects (Puschner, 2018). The alkaloid derives its name from *A. muscaria*, the species from which it was originally isolated in 1869. For many years thereafter, it was mistakenly regarded as the primary active component responsible for the mushroom’s effects (Shah, et al., 2009). From a pharmacological perspective, muscarine activates muscarinic acetylcholine receptors associated with parasympathetic signaling in a non-specific manner. Muscarinic acetylcholine receptors are expressed in smooth muscle and exocrine glands, allowing muscarine to influence multiple peripheral organs. The compound is not readily broken down by cholinesterase enzymes, which contributes to a longer duration of activity. Because its structure containes a permanently charged quaternary ammonium group, it cannot readily penetrate the blood–brain barrier and therefore does not directly affect the central nervous system (Flament, et al., 2020; Voynova, et al., 2020). In cases of *A. muscaria* intoxication, muscarine is mainly responsible for peripheral autonomic manifestations such as excessive salivation, increased sweating, slowed heart rate, and gastrointestinal disturbances. These effects are generally mild and transient, although substantial exposure may cause marked cholinergic toxicity. Overall, this compound contributes only secondarily to the mushroom’s toxic profile, whereas the central neuropsychiatric effects are primarily attributed to ibotenic acid and muscimol (Stoeva-Grigorova, et al., 2025).

Ibotenic acid is an alkaloid with neurotoxic and intoxicating properties and shows structural similarity to the neurotransmitter glutamic acid. After ingestion, a portion of ibotenic acid is metabolized into muscimol under the influence of gastric acid and heat (National Center for Biotechnology Information, 2024). Following oral ingestion, ibotenic acid is absorbed from the gastrointestinal tract and distributed to the central nervous system after crossing the blood–brain barrier via an active transport mechanism. Approximately 10%–20% of ibotenic acid undergoes conversion to muscimol in the gastrointestinal tract, while the remaining fraction is largely eliminated unchanged in urine within several hours (Stříbrný, et al., 2012). Although ibotenic acid is partially converted into muscimol after ingestion, the majority of both compounds are eliminated relatively rapidly, predominantly through urinary excretion. Fluctuations in the concentrations of these two substances may contribute to variations in central nervous system symptoms (Rampolli et al., 2021). Ibotenic acid acts as a potent agonist of NMDA receptors as well as metabotropic glutamate receptors of group I (mGluR1 and mGluR5) and group II (mGluR2 and mGluR3). In addition, it is also a weak agonist of AMPA and kainate receptors (Rang et al., 2012). Unlike muscarine, ibotenic acid can cross the blood–brain barrier via an active transport mechanism and therefore primarily affects the central nervous system. Ibotenic acid produces entheogenic effects in humans at doses between 50 and 100 mg. The psychoactive threshold dose of ibotenic acid has been reported to range between approximately 30–60 mg, although some sources indicate a range of 50–90 mg. These reported dose ranges refer primarily to estimated amounts of purified active compounds described in experimental and toxicological literature rather than standardized doses of fresh or dried mushroom material, as the concentrations of ibotenic acid and muscimol may vary substantially depending on mushroom origin, preparation, and processing methods. Peak symptoms of intoxication occur approximately 2–3 h after oral ingestion and may include visual distortions or hallucinations, loss of balance, muscle tremors, and altered sensory perception. These effects usually persist for 6–8 h depending on the dose taken (Waser, 1967; Michelot, et al., 2003; Carboué and Lopez, 2021; National Center for Biotechnology Information, 2024).

Another important compound contributing to the psychoactive effects of Amanita muscaria is muscimol. For muscimol, psychoactive effects have been observed at doses of around 6 mg, while other reports suggest a range of 7.5–10 mg (Waser, 1967; Michelot, et al., 2003). Overall, these dose estimates should be interpreted cautiously, as they are derived from heterogeneous experimental and toxicological sources and do not establish a reliable dose–response relationship for fresh or dried *A. muscaria* preparations. Muscimol acts as a highly potent activator of GABA receptors located on both presynaptic and postsynaptic membranes and is capable of efficiently penetrating the blood–brain barrier. After absorption, muscimol is distributed within the central nervous system and is primarily eliminated through renal excretion. By promoting chloride ion influx through receptor-associated channels, it induces neuronal hyperpolarization and leads to a reduction in overall neural excitability (Flament et al., 2020). The highly variable and sometimes contradictory clinical manifestations associated with *A. muscaria* may partly reflect the opposing neuropharmacological actions of ibotenic acid and muscimol. Whereas ibotenic acid acts predominantly as an excitatory glutamatergic agonist contributing to neuroexcitation, agitation, perceptual disturbances, and toxic effects, muscimol primarily exerts inhibitory GABAergic effects associated with sedation, altered consciousness, impaired coordination, and central nervous system depression. Variability in the relative concentrations of these compounds, influenced by mushroom composition and preparation methods such as drying or heating, may contribute to the substantial variability in psychoactive experiences and toxicological outcomes reported in both clinical observations and online user discussions. In addition to its strong activity at GABA-A receptors, muscimol has also been shown to interact with GABA-C receptor subtypes (Gussin, et al., 2006). In clinical settings, exposure to muscimol is associated with central nervous system depression accompanied by perceptual disturbances, including altered visual experiences and hallucinations. Affected individuals may also present with impaired coordination, instability of posture, and involuntary muscle twitching reflecting neuromuscular involvement which, in severe cases, may precede generalized convulsions, along with gastrointestinal symptoms such as nausea and vomiting (Moss & Hendrickson, 2019; Flament, et al., 2020). Table 2 presents the comparative pharmacological mechanisms, toxicological effects, and selected characteristics of ibotenic acid and muscimol associated with *A. muscaria*.

**TABLE 2:** Comparative characteristics and toxicological effects of ibotenic acid and muscimol.

Feature	Ibotenic acid	Muscimol
Chemical nature	Alkaloid with neurotoxic and intoxicating properties, structurally similar to glutamic acid	Important compound contributing to the psychoactive profile of amanita muscaria
Metabolic relationship	Partly metabolized into muscimol under the influence of gastric acid and heat	Formed from ibotenic acid during metabolism and processing
Absorption	Absorbed after oral ingestion from the gastrointestinal tract	Absorbed after oral ingestion
Blood–brain barrier penetration	Crosses the blood–brain barrier via an active transport mechanism	Efficiently penetrates the blood–brain barrier
Excretion	Predominantly eliminated through urinary excretion	Primarily eliminated through renal excretion
Main pharmacological mechanism	Acts as a potent agonist of NMDA receptors and metabotropic glutamate receptors	Acts as a highly potent activator of GABA receptors
Reported psychoactive dose	Approximately 30–60 mg, with some sources indicating 50–90 mg	Around 6 mg, with other reports suggesting 7.5–10 mg
Main reported effects	Visual distortions or hallucinations, loss of balance, muscle tremors, and altered sensory perception	Central nervous system depression, perceptual disturbances, altered visual experiences, and hallucinations
Toxic manifestations	Mainly central nervous system symptoms related to excitatory neuroactivity	Impaired coordination, instability of posture, involuntary muscle twitching, possible generalized convulsions, nausea, and vomiting
Duration of effects	Usually 6–8 h depending on the dose taken	Not specifically reported
Role in intoxication	Primarily contributes to central neuropsychiatric effects	Contributes to the characteristic neuropharmacological profile and clinical manifestations of intoxication

Minor constituents such as muscazone, a photodegradation product of ibotenic acid, have also been reported in *Amanita muscaria*, although their toxicological relevance appears limited (Bhambri, et al., 2022). In addition to the major neuroactive compounds, several minor metabolites have been detected in *A. muscaria*, including compounds such as (R)-4-hydroxypyrrolidone derivatives, choline, acetylcholine, betaine, and various nucleoside-related metabolites, although their contribution to the toxicological profile of the mushroom appears to be negligible (Michelot & Melendez-Howell, 2003). Taken together, the combined activity of ibotenic acid and muscimol, along with the minor contribution of muscarine, underlies the characteristic neuropharmacological profile and clinical manifestations associated with *A. muscaria* intoxication. Overall, the pharmacokinetic profile of ibotenic acid and muscimol is characterized by gastrointestinal absorption, central nervous system distribution following blood–brain barrier penetration, partial metabolic conversion, and predominantly renal elimination.

## 4 Patterns of use and reported adverse health outcomes associated with *Amanita muscaria* consumption

In recent years, interest in the consumption of *A. muscaria* has increased, both in the context of recreational use and because of its alleged therapeutic properties promoted on social media. This growing trend has been accompanied by an increasing number of reports describing patterns of use as well as adverse health outcomes associated with the ingestion of this species. An analysis of cases of *A. muscaria* and *Amanita pantherina* poisoning hospitalized in the Department of Toxicology in Poznań showed that symptoms typically appeared within 30 min to 2 h after ingestion and included, among others, vomiting, hallucinations, psychomotor agitation, and depression of the central nervous system. The most serious complication observed during poisoning was acute respiratory failure (Łukasik-Głębocka et al., 2011). This complication may be related, at least in part, to central nervous system depression mediated by the potent GABAergic activity of muscimol, which may impair respiratory drive in severe intoxication. Comparative studies of *A. muscaria* and *A. pantherina* poisoning additionally showed that confusion and agitation were more frequently observed after ingestion of *A. muscaria*, whereas poisoning with A. pantherina more often resulted in loss of consciousness and coma (Vendramin & Brvar, 2014).

Several additional case reports describing *A. muscaria* poisoning have also been published. In a study describing nine cases of poisoning with mushrooms of the genus *Amanita* in children, including one case following the ingestion of *A. muscaria*, symptoms appeared within 30–180 min and mainly involved disorders of the central nervous system, such as ataxia, disturbances of consciousness, hallucinations, and seizures. A rapid and complete recovery was observed in all patients (Benjamin, 1992). Case reports also indicate the possibility of neuropsychiatric symptoms following the recreational consumption of this species. In one report, five individuals aged 18–21 consumed dried fruiting bodies of *A. muscaria* in order to induce hallucinations. Visual and auditory hallucinations occurred in four of them, and one person lost consciousness. After several days of observation, the patient was discharged without further complications, and the remaining participants did not report further complaints (Satora et al., 2005). In the journal Wiener Klinische Wochenschrift, a case was described of a 48-year-old man who, after consuming mushrooms mistakenly identified as *Amanita caesarea* but in fact *A. muscaria*, initially experienced vomiting and loss of consciousness. Approximately 18 h after ingestion he developed paranoid psychosis with visual and auditory hallucinations that persisted for 5 days and then completely resolved (Brvar et al., 2006). Further reports include cases of more severe poisoning. Among them was a young man who fell into a coma after consuming *A. muscaria*. Following rapid identification of the poisoning, gastric lavage, and symptomatic treatment, the symptoms resolved and the patient was discharged from the hospital on the third day of hospitalization (Mikaszewska-Sokolewicz et al., 2016). Two cases of severe toxicity following the ingestion of *A. muscaria* have also been described in the literature, including one resulting in the death of a 44-year-old man after ingesting several dried mushroom caps for psychoactive purposes. The second case involved a 75-year-old man who accidently consumed a foraged mushroom and made a full recovery after hospital treatment (Meisel et al., 2022). Another case report published in the European Journal of Case Reports in Internal Medicine in 2021 describes a patient who fell into a coma after the accidental ingestion of *A. muscaria*. Due to the rapid identification of the mushroom species and the implementation of symptomatic treatment, the symptoms resolved and the patient was discharged from the hospital on the fourth day after ingestion (Rampolli et al., 2021). In 2023, a case was described in the United Kingdom of a woman who developed acute delirium after ingesting *A. muscaria* to obtain an anxiolytic effect. After symptomatic treatment, the patient fully recovered and was discharged from the hospital after 24 h (Maung et al., 2023). In 2018, a man in Minnesota was also hospitalized after consuming wild mushrooms he had collected near his workplace, mistaking them for an edible species known from his native Burma. The mushrooms were identified as *A. muscaria* var. *Guessowii*, containing ibotenic acid and muscimol, which can cause neurological and gastrointestinal symptoms. The patient required intensive treatment but ultimately recovered. This event highlighted the risk of poisoning among immigrants accustomed to foraging mushrooms in their countries of origin and the need for education regarding local mushroom species (Taylor et al., 2019). Recent reports also indicate an increasing number of poisonings associated with the recreational consumption of this species. In November 2025, two simultaneous cases of acute *A. muscaria* poisoning were described in an elderly married couple, a 79-year-old man and a 78-year-old woman, who had consumed wild mushrooms collected and prepared at home. Symptoms appeared approximately 30 min after ingestion and included somnolence, nausea, severe vomiting, and rapidly progressing disturbances of consciousness leading to stupor and coma (Stoeva-Grigorova, 2025). In the same year, four cases of intentional *A. muscaria* poisoning related to recreational use were reported in Lithuania. The patients presented with symptoms including tremors, dizziness, respiratory failure, and paranoia, however all were discharged from the hospital in stable condition (Savickaitė et al., 2025).

In response to the growing trend of *A. muscaria* consumption, numerous profiles and posts promoting its alleged health benefits have appeared on social media. On various online platforms, presumed therapeutic benefits of this species are presented, often without support from reliable scientific data (Ordak et al., 2023; Pieta-Chrystofiak and Brohs, 2023). Current claims regarding the therapeutic benefits of Amanita muscaria are based predominantly on anecdotal reports, self-reported experiences, and observational social media data, while controlled clinical studies confirming efficacy and safety are currently lacking. Authors of such materials sometimes refer to unverified reports of clinical studies and publish recipes for various preparations made from *A. muscaria*, which are claimed to reduce the content of toxic compounds such as muscimol and ibotenic acid, thereby making the mushroom safe for consumption. The dissemination of such information on the Internet may increase health risks among users.

In 2023, results of studies analyzing consumer motivations, preferred forms of *A. muscaria* consumption, and the most frequently reported adverse effects associated with its consumption were published. The analysis included 5,600 comments and responses from 684 Facebook users, including 277 women and 407 men. The results indicated that motivations related to the consumption of this mushroom species may differ depending on sex. Women most frequently declared the use of *A. muscaria* to alleviate pain and skin problems, whereas men mainly indicated the desire to reduce symptoms of depression, insomnia, and stress. The most commonly reported adverse effects among women were headaches, whereas among men they included nausea, vomiting, headaches, and somnolence. The most frequently used forms of the mushroom were dried material and tinctures (Ordak et al., 2023). These findings should be interpreted cautiously, as they were based on self-reported data derived from social media users and were not validated in controlled clinical settings. Another study focused on participants of online discussion groups and analyzed both cultural and sociodemographic aspects related to the consumption of *A. muscaria*. The study included 95 participants, including 32 women, 60 men, and 3 individuals identifying with another gender, who were classified as either experimental users or regular users of this mushroom. The most frequently indicated reasons for consuming *A. muscaria* were presumed therapeutic effects, spiritual needs, enhancement of cognitive abilities, and curiosity. Less frequently reported factors included relaxation, pleasure, personal problems, and social pressure. Participants most often declared the use of dried material or specially prepared extracts, while their attention focused mainly on potential positive effects and reported little attention to possible health risks (Pieta-Chrystofiak and Brohs, 2023). The reported motivations and perceived benefits reflect subjective user experiences and do not constitute clinical evidence of therapeutic efficacy. Earlier survey studies conducted in online communities indicate similar user motivations. Data collected through online questionnaires distributed on Facebook and internet forums dedicated to mushrooms showed that the main reasons for consuming *A. muscaria* included potential pain relief and the alleviation of symptoms of depression, anxiety, insomnia, and chronic fatigue (Feeney, 2020).

In summary, the presented data indicate that the consumption of *A. muscaria* may lead to a wide spectrum of clinical symptoms, including both neurological and psychiatric disturbances as well as serious complications requiring hospitalization. The analysis of available publications also shows that poisoning with this species may result from both accidental ingestion and its intentional use for recreational or presumed therapeutic purposes. At the same time, the growing popularity of *A. muscaria* on social media and the easy access to unverified information regarding its use may contribute to an increase in such cases. Many publications emphasize that the dissemination of unverified content concerning the properties of this mushroom may constitute a significant public health risk. Therefore, further research on the mechanisms of *A. muscaria* toxicity and increased public awareness of the potential risks associated with its consumption are necessary.

## 5 Limitations of current knowledge, future research directions and public health implications

In recent years, an increasing trend in the consumption of *A. muscaria* can be observed. After the closure of shops selling various types of NPS, people began to look for simpler ways to intoxicate themselves as well as alternative self-treatment approaches for various health conditions (Ordak, et al., 2023). According to data published in the American Journal of Preventive Medicine, the number of Google searches for the term *A. muscaria* increased by as much as 114% between 2022 and 2023 (Leas, et al., 2024). Supporters of consuming *A. muscaria* explain this phenomenon by the lack of robust scientific evidence confirming the safety or risks associated with the use of various prepared forms of this mushroom, namely, dried material, tinctures, ambrosias and ointments. They believe that processing *A. muscaria* reduces the risk of adverse effects by removing toxic components. In social media they frequently claim that most of the research results published so far regarding the effects of *A. muscaria* on human health concern the consumption of raw fruiting bodies of this species. However, many of these claims remain unsupported by high-quality clinical evidence, and currently available data are derived mainly from case reports, experimental studies, and observational user-reported experiences. Studies published so far indicate that muscarine, due to its thermostability, is not destroyed during cooking (Chan, et al., 2023). A study comparing different *A. muscaria* extracts showed that the processing method may influence the content of ibotenic acid and muscimol. In the analyzed standardized water-ethanol extract, very small or negligible amounts of these compounds were found. However, a limitation of the study was the small number of analyzed preparations and the lack of direct comparison with concentrations in unprocessed material, which limits the ability to accurately assess the impact of processing on the content of ibotenic acid and muscimol (Dushkov, et al., 2023).

During the drying of *A. muscaria*, part of the ibotenic acid undergoes decarboxylation to muscimol, which leads to a decrease in the concentration of this acid. During cooking, a reduction in the amount of ibotenic acid and an increase in muscimol were also observed, particularly under acidic conditions, however short cooking did not cause a significant reduction in the total amount of toxins. In turn, cooking or soaking the mushrooms in water resulted in the rapid transfer of both compounds into the water (Tsunoda, et al., 1993). The method of processing *A. muscaria* may also affect the concentration of toxic elements such as cadmium and lead, which was highlighted in the most recent article published in 2025. The highest concentrations of lead were recorded in a tincture prepared directly from the raw material, whereas lower values were observed in water-based preparations such as decoction, infusion or cooked form. In contrast, cadmium concentrations were lower in decoctions and fermented preparations compared with dried samples. Although some preparation methods reduced the content of heavy metals, none of the analyzed preparations can be considered completely safe when consumed frequently or in excessive amounts (Ordak, et al., 2025).

Further research on the safety of its various processing methods is necessary. In particular, experimental studies analyzing changes in the concentrations of key active compounds such as ibotenic acid, muscimol and muscarine during different preparation processes are needed, including drying, cooking, fermentation and the preparation of alcohol and water extracts. It is also important to compare the concentrations of these substances in processed preparations with their content in raw fruiting bodies. Further studies should also include the analysis of the impact of processing on the content of heavy metals, which may constitute an additional health risk. Toxicological and pharmacological studies assessing the effects of different *A. muscaria* preparations on the human body are also necessary. The obtained results could provide reliable scientific data necessary for assessing health risks and developing recommendations regarding the safety of using preparations derived from this mushroom.

Another recommended measure is the introduction of appropriate legal regulations regarding the use of *A. muscaria*. In many countries, such as the United States, this mushroom is permitted, with the exception of some states, for example, Louisiana, which introduced a ban in 2005 (Louisiana State Legislature, 2025). Another country recognizing *A. muscaria* as a controlled substance is the Netherlands (Ministerie van Binnenlandse Zaken en Koninkrijksrelaties, 2023) as well as Romania (ICEERS, 2026). In Thailand, mushrooms producing hallucinogenic effects have been classified as controlled substances in category V, therefore their use and distribution are prohibited there (Thailand Law Forum, 2011). In Japan, these two mushroom species can be purchased without major difficulty. They are offered both online and in specialized shops, usually in the form of dried fruiting bodies or mushroom powder (Gonmori, et al., 2012). Many products containing *A. muscaria* or muscimol are available on the market and are offered as food or dietary supplements, even though they do not have official safety recognition by the FDA (U.S. Food and Drug Administration, 2024). These ingredients do not have GRAS status (Generally Recognized as Safe–a substance generally recognized as safe) nor an NDI notification (New Dietary Ingredient–a new dietary ingredient), and in light of reports of serious adverse effects their sale may violate regulations intended to protect public health. Although many manufacturers declare control of the content of active substances and contaminants in their products, there are currently no widely recognized testing standards or reference points allowing the assessment of the concentration and potency of muscimol. Additionally, it is therefore necessary to develop and standardize analytical methods enabling reliable determination of muscimol and ibotenic acid concentrations in various *A. muscaria* preparations.

It is necessary to introduce clear regulations regarding the production and sale of products containing *A. muscaria* and muscimol, including age restrictions, maximum doses, appropriate labeling and analytical standards ensuring control of their composition and safety. At the same time, the need for educational activities and the involvement of mental health professionals in informing about the risks associated with the use of these products is emphasized, as unlike some other psychoactive substances they do not have confirmed therapeutic applications (Leas, et al., 2024). In the Scientific Memorandum: *A. muscaria* (9 September 2024) published by the FDA, it was assessed whether the use of this mushroom in food meets the GRAS criteria. The document states that due to the lack of sufficient safety data and the available information indicating potential harmfulness, *A. muscaria*, its extracts and active compounds (muscimol, ibotenic acid and muscarine) do not meet the criteria for being recognized as safe for use in food (Scientific Memorandum: *Amanita Muscaria*, 2024).

In summary, the legal status of *A. muscaria* is currently inconsistent and varies depending on the country, which leads to a situation in which in some states the sale of products containing this mushroom is restricted, while in others it takes place without significant control. At the same time, products containing *A. muscaria* are available on the market despite the lack of recognized standards for assessing their safety and clear regulations regarding their composition. Therefore, it is necessary to introduce clear regulations defining the rules for the production, distribution and sale of preparations containing this mushroom species. These regulations should include the obligation of laboratory control of composition, the determination of permissible levels of active substances and a system of supervision over the safety of such products. The introduction of consistent legal regulations could reduce the health risks associated with the uncontrolled availability of preparations containing *A. muscaria*.

Another recommendation is the introduction of targeted educational measures. An analysis of discussions among users of the Reddit platform indicates the need to apply more advanced research methods enabling a more in-depth analysis of content published on social media. This approach could provide more detailed information about user experiences and their perceptions related to the use of *A. muscaria* (Hartwig, et al., 2025). Research results indicate that school prevention programs can effectively increase the level of knowledge about psychoactive substances and improve decision-making skills and resistance to peer pressure. Programs based on the development of psychosocial skills proved to be particularly effective and also contributed to reducing the use of marijuana and other drugs. Interventions focused solely on providing knowledge also led to increased awareness of the risks associated with the use of psychoactive substances. These results indicate that educational activities may constitute an important element of prevention strategies aimed at reducing the use of psychoactive substances (Faggiano, et al., 2008). In response to the lack of prevention programs targeted at youth from Indigenous communities in Australia, the Strong & Deadly Futures program was developed, aimed at preventing the use of alcohol and other psychoactive substances and supporting the mental wellbeing of students. Its effectiveness is to be evaluated in a randomized study conducted in secondary schools, including the analysis of changes in the level of knowledge about psychoactive substances and behaviors related to their use. This indicates that the implementation of similar educational programs may constitute an important element of preventive measures, contributing to increasing young people’s knowledge about the risks associated with the use of psychoactive substances (Stapinski, et al., 2022).

Educational activities concerning *A. muscaria* should include the dissemination of reliable information about its pharmacological properties, potential adverse effects and the health risks associated with its use. The dissemination of knowledge in this area could contribute to increasing public awareness of the possible consequences of using this mushroom. Education could also limit the spread of unverified information regarding the alleged health benefits of *A. muscaria*. The introduction of information programs directed particularly at young people could support more informed decision-making regarding the use of psychoactive substances. Such activities could constitute an important element of health prevention aimed at reducing the potential harms resulting from the use of *A. muscaria*. Overall, the current body of evidence regarding *A. muscaria* remains methodologically heterogeneous, consisting predominantly of experimental pharmacological studies, clinical case reports, and observational digital data, with a lack of controlled prospective clinical research. Key limitations of current knowledge and recommended research and public health actions related to *A. muscaria* are summarized in Table 3.

**TABLE 3:** Key limitations of current knowledge and recommended research and public health actions regarding *Amanita muscaria*.

Area	Current limitations	Recommended actions
Toxicological evidence	Limited number of clinical and toxicological studies. Many reports based on case descriptions	Conduct further toxicological and pharmacological studies on *Amanita muscaria* and its active compounds
Processing methods	Insufficient data on how drying, cooking, fermentation and extraction influence ibotenic acid and muscimol concentrations	Experimental studies comparing muscimol and ibotenic acid concentrations in raw, dried, boiled, fermented, and extract-based *Amanita muscaria* preparations
Active compound variability	Lack of standardized methods for determining concentrations of muscimol and ibotenic acid in different preparations	Development and standardization of analytical methods, including LC–MS/MS, for reliable quantification of muscimol and ibotenic acid
Heavy metal contamination	Limited data on cadmium and lead concentrations in different *Amanita muscaria* preparations	Systematic studies assessing heavy metal content in various processing methods
Regulatory frameworks	Legal status of *Amanita muscaria* differs between countries and lacks consistent regulation	Development of clear regulations regarding production, labeling and sale of *Amanita muscaria* products
Public awareness	Widespread dissemination of unverified information regarding alleged therapeutic benefits on social media	Educational initiatives informing about pharmacological effects and potential health risks

## 6 Conclusion

The increasing interest in *Amanita muscaria* reflects a broader shift toward accessible psychoactive substances in response to changes in drug markets and regulatory controls affecting new psychoactive substances. Available evidence indicates that the mushroom contains neuroactive compounds, primarily ibotenic acid and muscimol, which are responsible for a wide spectrum of neurological and psychiatric manifestations ranging from mild perceptual disturbances to severe intoxication requiring hospitalization reported in cases of intoxication. At the same time, the growing promotion of its alleged therapeutic properties on social media and online platforms contributes to the dissemination of unverified information that may underestimate potential health risks. Although emerging reports suggest increasing intentional consumption of *A. muscaria* for recreational and self-therapeutic purposes, current evidence remains limited predominantly to case reports, observational studies, and user-reported experiences, while controlled clinical studies are lacking. Although emerging reports suggest increasing intentional consumption of *A. muscaria* for recreational and self-therapeutic purposes, current evidence remains limited predominantly to case reports, observational studies, and user-reported experiences, while controlled clinical studies are lacking. Current knowledge regarding the safety of different preparation methods and patterns of use remains limited, highlighting important gaps in toxicological and pharmacological data. Future research should therefore focus on the mechanisms of toxicity, the effects of various processing methods on active compound concentrations, and the development of reliable analytical and regulatory frameworks to better assess and mitigate potential public health risks associated with *A. muscaria*. The development of standardized analytical methods, clearer regulatory frameworks, and targeted public health education may also contribute to reducing the potential harms associated with the growing popularity of *A. muscaria*.
